# Case Report: Treatment of chondroblastoma of the first metatarsal bone in children with the induced membrane technique

**DOI:** 10.3389/fped.2025.1618704

**Published:** 2025-12-04

**Authors:** Zonghuan Li, Zheng Wang, Hong Liu, Shaobo Zhu

**Affiliations:** 1 Department of Orthopedics Trauma and Microsurgery, Zhongnan Hospital of Wuhan University, Wuhan, Hubei, China; 2 Department of Orthopedics, Xiaogan First People’s Hospital, Xiaogan, Hubei, China

**Keywords:** chondroblastoma, metatarsal, children, pediatric, induced membrane technique

## Abstract

**Introduction:**

Chondroblastoma occurring in the metatarsal bone is relatively rare clinically. Its diagnosis is confirmed by pathological examination, and the main treatment is lesion curettage and bone grafting. In this article, we report a case of chondroblastoma of the first metatarsal bone in a child treated with the induced membrane technique and review the relevant literature.

**Case presentation:**

A 9-year-old female patient presented with swelling and pain at the first metatarsal bone of the left foot one month ago without obvious cause. Imaging examination indicated local bone destruction with expansive growth in the first metatarsal. In the first-stage operation using the induced membrane technique, the lesion was removed and filled with bone cement. In the second-stage operation, the bone cement was removed and autologous fibula bone grafting was performed. Four months later, the bone at the metatarsal site healed well, the child could walk normally, and no tumor recurrence was observed during one-year follow-up. The bone at the fibula donor site also healed well, and no complications such as ankle instability at the donor site were seen.

**Conclusion:**

Chondroblastoma of the metatarsal bone in children is relatively rare. The two-stage operation of fibula transplantation using the induced membrane technique can effectivelybridge metatarsal bone defect.

## 1 Introduction

The incidence of chondroblastoma accounts for less than 1% of all primary bone tumors (1). It mostly occurs in people aged 5–25, with a male-to-female ratio of 2:1. Generally, it is accompanied by local swelling, pain, and obvious tenderness. It often occurs in the femur, humerus, and tibia. Bones of the hands and feet account for about 10% of the cases, but it is more common in the talus and calcaneus, and relatively rare in the metatarsal bones (2, 3). The x-ray shows an osteolytic lesion with surrounding sclerosis. The treatment method is surgical removal of the lesion and bone grafting. We report a case of chondroblastoma of the first metatarsal bone in a 9-year-old girl successfully treated with the induced membrane technique.

## 2 Case report

The patient is a 9-year-old girl. One month before admission, without trauma, the first metatarsal bone of her left foot became swollen and painful. The symptoms could be slightly relieved after rest but worsened after walking while bearing weight. An x-ray taken at a local hospital showed that the first metatarsal bone of her left foot was enlarged. She had been in good health previously and had no special medical history.

Physical examination revealed obvious swelling on the medial side of the left dorsal foot. The local skin was intact without any ulceration or tenderness, and the skin temperature was normal. When walking, due to pain on the medial side of the left foot, the patient dared not bear weight and presented with a mild limping gait. There was tenderness at the swollen area of the left foot. The flexion and extension of the hallux were slightly limited, while the movement of the remaining toes showed no obvious abnormalities. The blood circulation, and sensation of the distal parts of the other extremities were all good.

**Figure 1:**
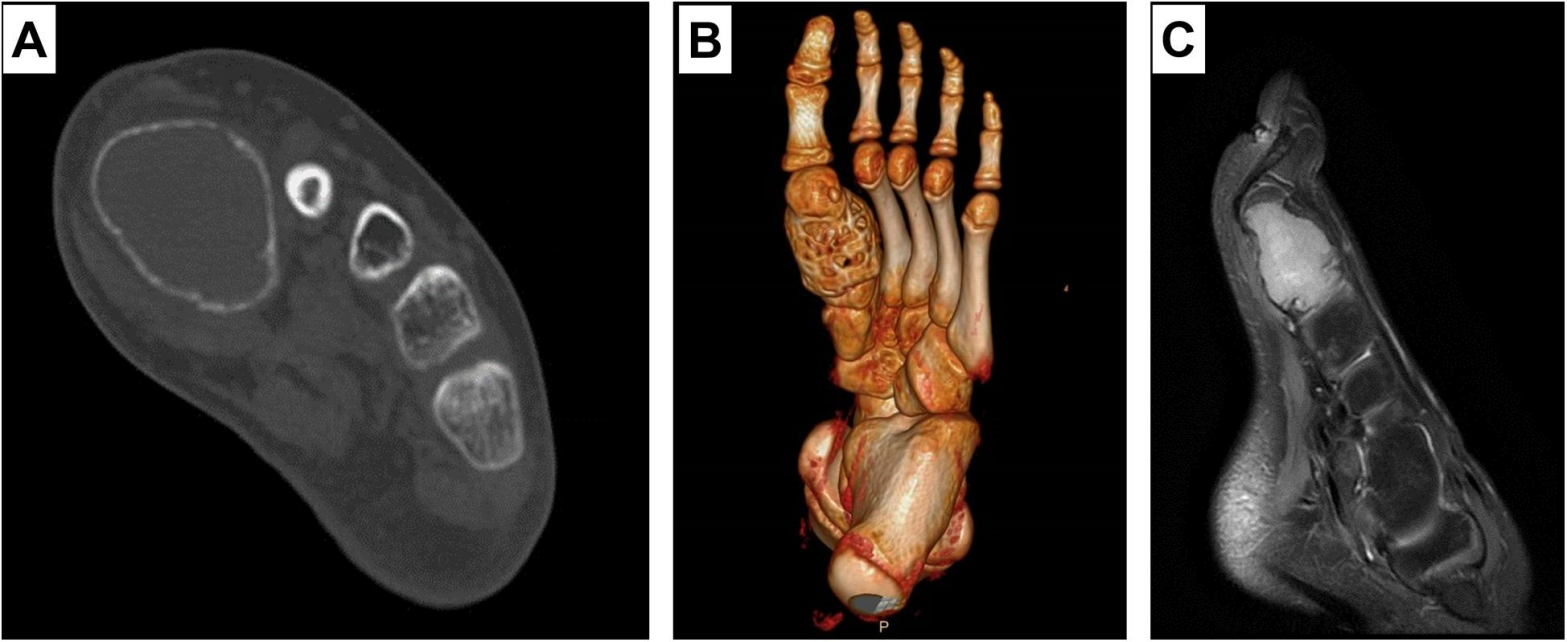
Preoperative CT **(A,B)** shows a space-occupying lesion in the first metatarsal bone, with expansile changes in the first metatarsal bone and thinning of the cortex; MRI **(C)** shows that the lesion invades the proximal articular surface of the first metatarsal bone, and a zone of bone edema can be seen at the distal end of the medial cuneiform.

After the patient was admitted to the hospital, relevant examinations were completed. The CT scan of the foot indicated an expansive bone destruction of the first metatarsal bone (Figures 1A,B), and the length of the bone defect was measured to be 4.2 cm. The MRI of the foot showed a high signal on the proton density (PD) sequence (Figure 1C) and a low signal on the T1-weighted image in the first metatarsal bone of the left foot. Considering the patient's age and the extent of the bone defect, a staged treatment with the induced membrane technique was planned.

**Figure 2:**
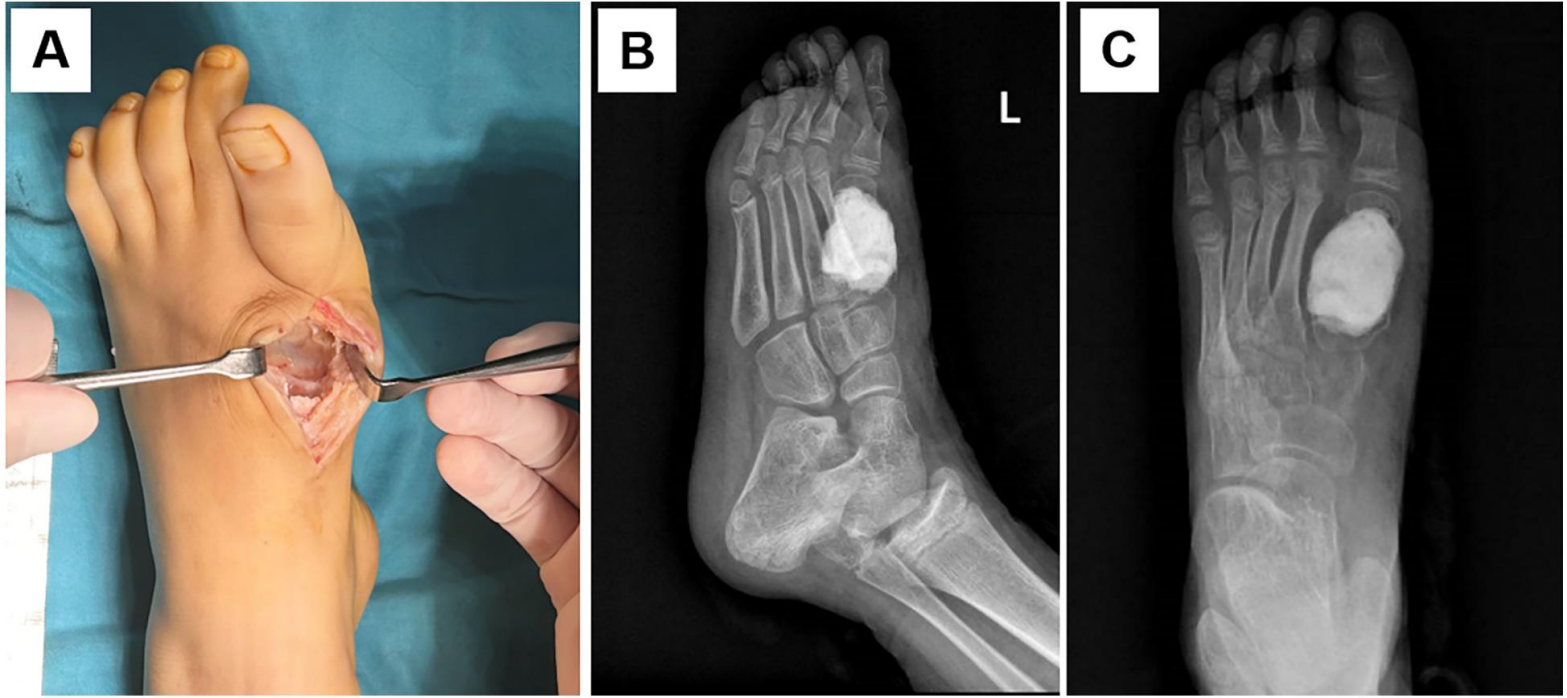
In the first—stage surgery of the induced membrane technique, it was observed that the lesion involved the proximal articular surface of the first metatarsal bone **(A)**. During the operation, the lesion was removed and bone cement was filled **(B,C)**.

**Figure 3:**
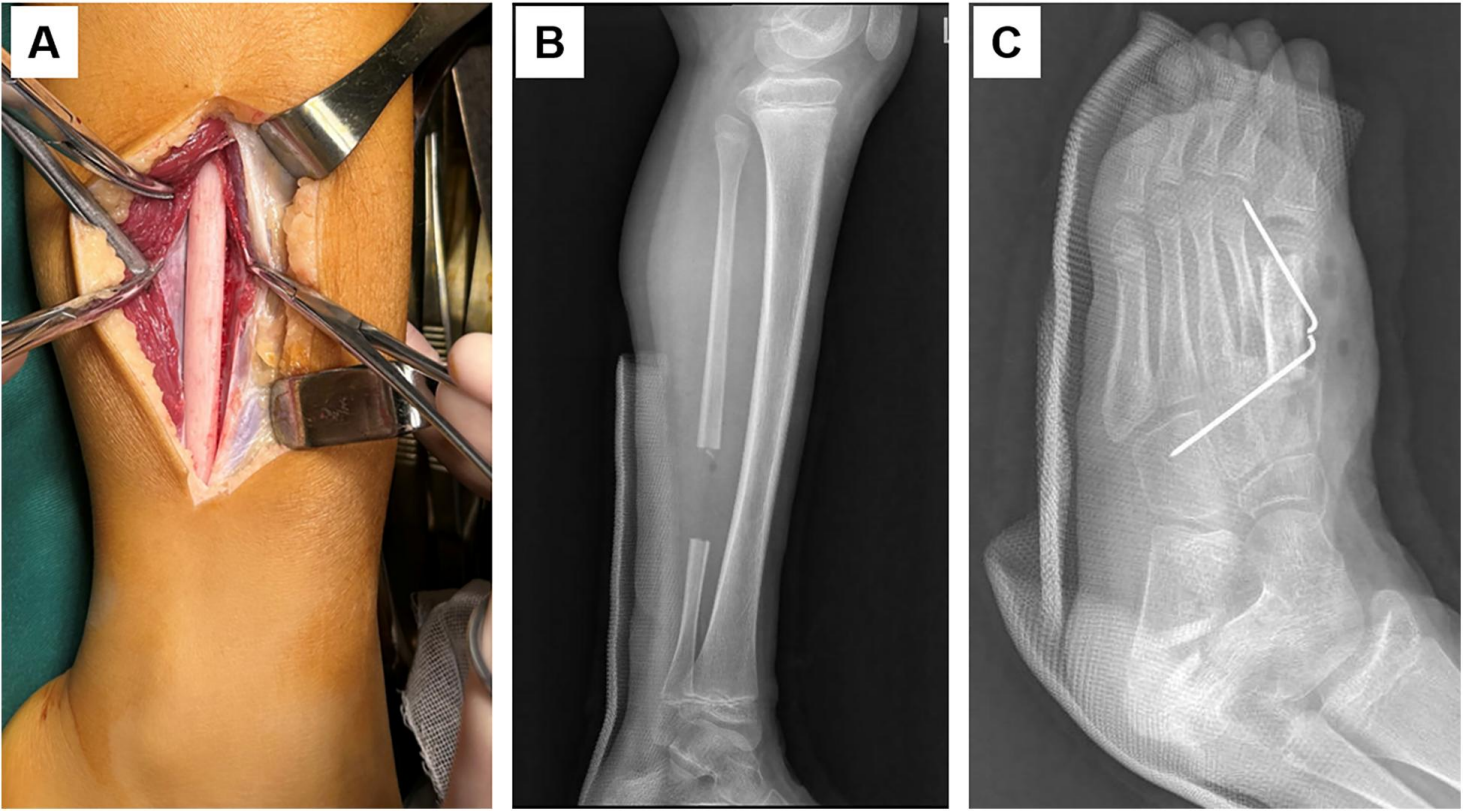
When harvesting the fibula, the integrity of the external periosteum of the fibula was preserved **(A)**. In the second-stage surgery of the induced membrane technique, the bone cement was removed, and a fibular bone graft was performed **(B,C)**.

**Figure 4:**
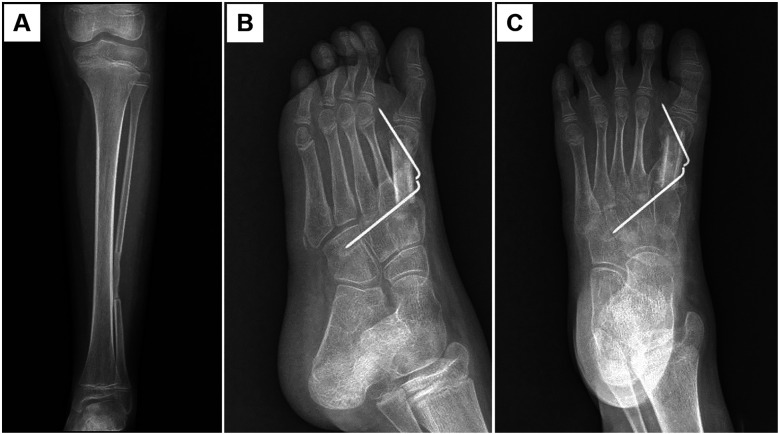
Two months after bone grafting, new bone formation was observed in the fibula at the donor site **(A)**, and the bone at the first metatarsal healed well **(B,C)**.

In the first-stage operation, a resection of the metatarsal lesion and filling of the bone cavity with bone cement were performed (Figure 2). The postoperative pathological examination reported chondroblastoma. Six weeks later, the second-stage operation was carried out, including the removal of the bone cement, autologous fibular bone grafting and internal fixation, and plaster immobilization (Figure 3). An incision was made on the medial aspect of the dorsum of the left foot to expose the metatarsal bone defect. The induced membrane was incised longitudinally while preserving its integrity. The bone cement was removed, and the medullary cavities on both sides were unblocked. An autologous fibula was implanted into the bone defect and fixed with 1.5 mm Kirschner wire. A cast was applied for immobilization for one month after the operation. Partial weight-bearing was allowed gradually after callus formation was observed at 2 months postoperatively. Antibiotics was administered prophylactically 30 min before surgery, and continued to be used within 24 h after the surgery. Analgesics were used according to the pain condition after the operation. Two months after the operation, new bone formation was observed at the fibular donor site (Figure 4A), and bone callus was visible at the bone graft site (Figures 4B,C). Four months later, the newly-formed fibula was nearly normal. The child could walk normally. No tumor recurrence was observed during one-year follow-up. The bone at the fibula donor site also healed well, and no complications such as ankle instability at the donor site were seen. The patient's pathological examination result was benign. The surgical wound and bone healed smoothly, and the fibula in the donor area regenerated. The patient's guardians were satisfied with the treatment and rehabilitation process. The timeline diagram of clinical events and surgical interventions was showed in Supplementary Figure S1.

## 3 Discussion

Non-vascularized bone grafting can be used for small-scale bone defects. However, when the bone defect exceeds 5 cm, the risk of absorption of the non-vascularized bone graft increases (4). Commonly used treatment methods include vascularized bone grafting, the Ilizarov technique, and the membrane induction technique. Vascularized bone grafting requires high-level microsurgical skills, and the delicate blood vessels in children further increase the surgical risk. The Ilizarov technique has limitations such as a long period of wearing an external fixator, as well as problems like pin tract infection and joint stiffness. The induced membrane technique is relatively simple. Although it is a two-stage operation, the first-stage operation can precisely clarify the nature of the lesion without adding extra surgeries or costs, making it a good choice for surgical treatment. In this case, as the patient was a 9-year-old girl, we chose the safer and simpler staged induced membrane technique.

In 1986, French scholars Masquelet et al. (5) first used the combination of the induced membrane and autologous bone grafting to successfully treat a large bone defect measuring 25 cm in length. Subsequently, this technique has been widely applied in the treatment of infected bone defects, aseptic non-unions, and bone defects after tumor resection, achieving favorable results. The results of a goat 3 cm bone defect animal experiment show that bone grafting within the induced membrane can successfully repair the bone defect, while simple bone grafting or having only the induced membrane alone is ineffective for bone repair (6). Basic research shows that the induced membrane is a highly vascularized tissue that can secrete a large number of cytokines and participate in local biological processes. The expression of VEGF, TGF-β1, and BMP-2 in the induced membrane tissue increases, promoting angiogenesis and osteogenesis (7, 8). This technique is particularly suitable for treating bone defects after the resection of bone tumors, with a treatment success rate of 87.5% (9).

We used the induced membrane technique to treat this case for two reasons. On the one hand, compared with needle biopsy, routine pathological examination after lesion curettage has a higher degree of certainty/accuracy. On the other hand, the induced membrane can promote the bone healing of traditional bone grafting locally and avoid treatment failure caused by bone dissolution/absorption. Moreover, given the 4.2 cm defect size, curettage rather than biopsy was adopted. Conversely, if a needle biopsy is performed first to clarify the nature of the lesion before the induced membrane technique, three surgeries are required: biopsy, tumor resection with bone cement filling, and bone grafting. However, our method combines the first two surgeries into one, achieving the same therapeutic effect in a shorter time with fewer operations.

In addition, several techniques in this operation are crucial for treatment. When harvesting the fibula from the donor site, to maintain the stability of the ankle joint at the donor site, at least 5 cm of the distal fibula should be retained. When dissecting to the periosteum layer, the periosteum should be incised longitudinally and its integrity should be preserved. After the fibula is removed, the periosteum should be sutured intact with absorbable sutures. When grafting bone at the recipient site, small holes should be drilled on the surface of the fibula and the cortical surface should be roughened to facilitate the ingrowth of surrounding bone. In the case we reported, the donor site completely regenerated. While achieving the repair of the recipient site, there were no complications at the donor site. Deventer reported 38 cases of chondroblastoma, with a male-to-female ratio of 2:1 and ages ranging from 11 to 51 years. Intralesional curettage followed by bone grafting after treatment with hydrogen peroxide can reduce the recurrence rate (10). Our experience is that after curettage of the lesion, the cyst wall of the lesion is wiped with dry alcohol cotton balls, rinsed with normal saline, and then cauterized with an electrocautery. Bone cement generates heat during the curing process, with temperatures reaching 60–70°C, which can kill local residual tumor cells and can also be used as a local adjuvant treatment. The purpose of different treatment methods is to kill residual tumor cells and reduce the recurrence rate.

Usually, autologous iliac bone grafting is used in the induced membrane technique (11). Cancellous bone provides a much greater surface area for new bone deposition than cortical bone. Generally, in cortical bone autografts, the rate of replacement of the graft bone by newly formed bone is much slower. However, in this case, we used fibular bone grafting instead of iliac bone grafting. On one hand, the surface of the iliac bone in children is covered with cartilage. On the other hand, cortical bone grafts can provide structural support for a period of time, which is necessary when the bone defect is extensive (such as in the treatment of bone tumors). Moreover, studies have shown that the application of free non-vascularized fibular graft in the induced membrane technique can shorten the healing time and improve the final outcome (12, 13).

Chondroblastoma of the metatarsal bone in children is relatively rare, and this case increases the sample size of such cases in the current literature. Moreover, for such bone defects, the reconstruction methods vary depending on the scope of the bone defects. There are various methods for reconstructing metatarsal defects: no bone grafting (14), bone chip (15), autologous ilium (16), or autologous fibula (17), etc. The case report by Florio M et al. (18) describes a 9-year-old with giant cell tumor (GCT) in the 4th metatarsal, rare in pediatric patients. Treated with wide resection, phenol adjuvant therapy, and Y-shaped reconstruction using non-vascularized fibular graft with Kirschner-wire fixation, the patient recovered fully with 2-year follow-up showing no recurrence and good function. Another retrospective study (19) analyzed 10 pediatric cases using non-vascularised fibular autograft for non-traumatic bone defects. With 63-month mean follow-up, 8 achieved union in 28 weeks on average. Four had graft fractures, four infections. It's effective for reconstructing pediatric bone defect with non-vascularised fibular autograft. Rengsen P (20) describes a 14-year-old girl with GCT in the 2nd metatarsal treated with en-bloc resection and reconstruction using a non-vascularised fibular graft fixed with a dynamic compression plate. At 24-month follow-up, the graft united, with good joint function, no recurrence, and high AOFAS scores. Thus, in this case, we repaired the defect by autologous fibula.

Chondroblastoma occurring in the metatarsal bones of children is extremely rare. Tosyalı HK (21) reported the treatment of 38 cases of chondroblastoma. Among these 38 cases, the most common location was the proximal femur, and there was no report of the tumor occurring in the metatarsal bones. Laitinen reported 177 cases of chondroblastoma. 15% of them occurred in the foot, but none of them occurred in the metatarsal bones (22).

In another study (15), a total of 104 cases of chondroblastoma were reported. Thirteen cases occurred in the feet, with only one case involving the metatarsal bone, and the rest occurred in the talus or calcaneus. Davila JA (23) retrospectively analyzed 25 pathologically confirmed cases of chondroblastoma of the hands and feet. The average age of the patients was 23 years old, and 3 cases occurred in the metatarsal bones. We reviewed Chinese and English literature and searched for cases of chondroblastoma of the metatarsal bone. Only a few scattered reports were found, and such cases were rare in children (Table 1). Only one study has reported a case of chondroblastoma of the metatarsal in a child. In this case, imaging showed a 7 mm diameter cystic lesion with calcification. The patient was treated with curettage, and due to the small size of the lesion, no bone grafting was performed. At the 24-month follow-up, there was no recurrence.

**Table 1:** Reported cases of chondroblastoma of the metatarsal bone in the literature.

Report	No. of cases	Age	Location	Size of bone defect	Treatment	Type of bone graft
Dhatt SS (17)	1	20 yrs	2nd metatarsal	10 cm*5 cm	Curettage + autologous fibula bone graft	Autologous structural fibular bone graft
Prohaska DJ (14)	1	14 yrs	2nd metatarsal	7 mm	Curettage alone	No bone graft
Bloem JL (15)	1	70 yrs	2nd metatarsal	Not reported	Curettage + bone graft, amputation after recurrence	Bone chip
Abdelwahab IF (24)	1	27 yrs	4th metatarsal	Not reported	Curettage + bone graft	Not reported
Ozkurt B (25)	1	23 yrs	4th metatarsal	3 cm*2 cm*4.5 cm	Curettage + bone graft	Not reported
Kobayashi S (16)	1	34 yrs	1st metatarsal	Not reported	Curettage + bone graft	Autologous bone grafting from the ilium
Atalar H (2)	1	23 yrs	Unknown	Not reported	Curettage + bone graft	Not reported

This case report has certain limitations. Due to the rarity of the case, this paper only reports one case, and the follow-up time is relatively short. It is still unknown whether long-term recurrence will occur, or whether complications such as metatarsal growth and development disorders and foot deformities will appear. We will continue to follow up this case in the later stage.

## 4 Conclusion

Chondroblastoma of the metatarsal bone in children is relatively rare. The two-stage operation of fibula transplantation using the induced membrane technique can effectivelybridge metatarsal bone defect and can be considered as a treatment option.

## Data Availability

The original contributions presented in the study are included in the article/Supplementary Material, further inquiries can be directed to the corresponding authors.

## Supplementary material

Supplementary Figure S1 The timeline of clinical events and surgical interventions.
