# Supplementary figures and images for: Case Report: Treatment of chondroblastoma of the first metatarsal bone in children with the induced membrane technique
Source: Front Pediatr. 2025 Dec 4;13:1618704. doi: 10.3389/fped.2025.1618704 (PMC12711719; doi:10.3389/fped.2025.1618704)

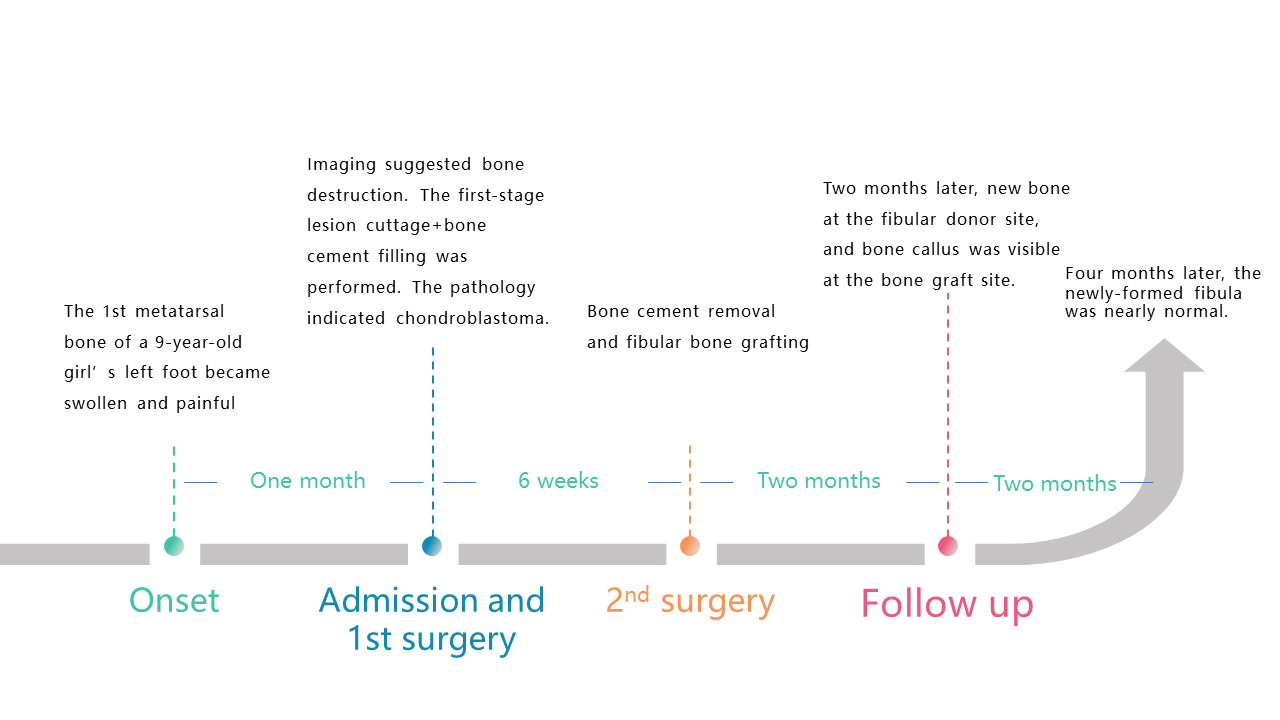

Supplement: Supplementary Figure S1 — The timeline of clinical events and surgical interventions. [file Image1.tif]
